# Social Accounting as an Enabling Tool to Develop Collective Organizational Citizenship Behavior in the Diocese of Bilbao

**DOI:** 10.3389/fpsyg.2020.00077

**Published:** 2020-02-14

**Authors:** Jose Luis Retolaza, Ricardo Aguado, Leire San-Jose

**Affiliations:** ^1^Economic Department, HUME, University of Deusto, Bilbao, Spain; ^2^Financial Economics II, ECRI, University of the Basque Country, UPV/EHU, Bilbao, Spain

**Keywords:** social accounting, organizational citizenship behavior, religious organizations, social organizations, theory of human action, transcendent motivation social performance, stakeholder theory

## Abstract

Religious oriented organizations (ROOs) have frequently higher levels of motivation among their employees, because the aims of ROOs and those of collaborators and stakeholders are usually aligned. However, sometimes, when the management of ROOs becomes professionalized, tensions between aims and efficiency are more frequent, and productivity levels start to decline. The most widespread current management theories are focused on profit maximization and are not especially helpful to religious organizations which try to enhance their productivity levels and, at the same time, achive their mission and aims. In order to fill this gap, in this research, we will develop two main concepts: social accounting and organizational citizenship behavior (OCB). We will propose the use of social accounting to calculate the social value generated by ROOs and, from that point, build new indicators able to measure the organizational citizenship behavior (OCB) of collaborators working in ROOs. We will exemplify this theoretical development with the actual case of the diocese of Bilbao. In short, the main objectives of this work are two. The first objective is the development of a theoretical framework able to enhance the levels of social value creation inside religious (and socially oriented) organizations using social accounting. The second objective is the use of data from the 16 educative centers of the diocese of Bilbao to ilustrate that social accounting is a valid tool to measure social value. Additionally, we will show that social accounting can be a tool to assess management decisions in order to enhance organizational and individual OCB in ROOs and, in this way, generate moral satisfaction for employees and collaborators in their organizations.

## Introduction

Social entities with a religious orientation usually have an aim that transcends them and is linked with the desire of helping people in need as a result of professing a particular faith. This transcendent aim is shared frecuently by employees and other stakeholders which are heavily committed to the objectives of the organization and, therefore, show a high productivity level.

However, as this kind of organization becomes more professionalized, it is normal for the aforementioned productivity level to decline. As stated in the prospect theory by Kahneman and Tversky (1979), when managers and employees have to take decisions under uncertainty, they underestimate decisions that lead to probable (but not completely certain) results, whereas they tend to focus on decisions that lead to solutions that will be achieved with total certainty. This trend, also called the certainty effect (Tversky and Kahneman, 1989), can damage the performance of organizations that are devoted to a mission because social results are much more uncertain compared to economic results. This tension between the aim of the organization and economic results (which are instrumental resources) creates conflicts both at the personal and institutional levels between efficiency/productivity (linked to the optimal use of resources) and effectiveness (achieve the goals of the organization) inside the organization. The most widespread current management theories are focused on profit maximization and are not especially helpful to religious organizations which try to enhance their productivity levels and, at the same time, achieve their mission and aims. In order to fill this gap, in this research, we will develop two main concepts. The first one is organizational citizenship behavior (OCB). OCB is understood as the value generated by an organization above the resources that have been utilized. The second concept is social accounting, which allows the quantification of the value generated by an organization beyond the mere market value. The linkage between these two concepts plus the motivational proceses of employees and stakeholders participating in religious organizations will be structured through the theory of human action (Pérez López, 1991). This framework will make possible an original understanding of individual OCB.

This research will develop the case of the diocese of Bilbao (Bilbao is the largest metropolitan area of the Basque region, located in the north part of Spain). The diocese has started a process of incorporating social accounting to its management system. The first step has been to implement social accounting to its 16 educative centers. The second step, currently under development, is to measure the social value that the diocese is generating through its almost 300 parishes and *Caritas* services. The interest of the diocese of Bilbao in incorporating social accounting in its management system is threefold. In the first place, there is an interest to communicate to the rest of society and major stakeholders (families, members of the Church, public administration, society as a whole) the market value, social value, and emotional value generated by each and all parts of the diocese. Social accounting can be a valid tool to identify and calculate in an objective way those values. Secondly, the diocese is interested in improving its strategic planning and the overall management system. The monetization (in euros) of the social and emotional value generated by the diocese makes easier the incorporation of social objectives into the strategic planning and the management system. In this way, benchmarking processes and the use of the balanced scorecard can be applied to all activities of the diocese. In the third place, the diocese is interested in facilitating feedback and accurate information about its activities and results to its network of employees, volunteers, and collaborators (onwards, we will refer to this network simply as collaborators). This information about the value generated to other people (usually, people in need) by the diocese may have a positive influence in the motivation of the network of collaborators. This third perspective, linked with accurate feedback, motivation, and empowerment of collaborators will be the one developed further in this research.

In short, the main objectives of this work are two. The first objective is the development of a theoretical framework able to enhance the levels of social value creation inside religious (and socially oriented) organizations using social accounting. The second objective is the use of data from the 16 educative centers of the diocese of Bilbao to ilustrate that social accounting is a valid tool to measure social value and assess management decisions in order to enhance it. After this first introductory section, the paper will develop the concept of organizational citizenship behavior (OCB) in its second section. In the third section, anthropological and social bases of OCB will be analyzed. In the fouth section, the case of the diocese of Bilbao will be explained. A last fifth section with conclusions will close this work.

In our view, religious organizations and/or organizations with a social aim need specific management tools to enhance their performance. This research is an effort trying to provide academics and practitioners with the necessary tools to do so. Hopefully, this work will open new lines of research focused in this kind of organization.

## Theoretical Framework: Understanding the Involvement of Collaborators With Social Entities of Religious Orientation Through Organizational Citizenship Behavior

Why do some people contribute more to their organizations than what is compulsory by contract? And, why those persons or others are committed to excellence, promoting it in their organizations without any form of explicit or implicit reward? This conduct, known as organizational citizenship behavior (OCB) is characterized by the use of decision-making rules that go beyond the maximization of the personal interest (Smith et al., 1983; Organ, 1988; Organ and Ryan, 1995; Podsakoff et al., 1996, 2000). OCB can be a useful tool to analyze and enhance the management of religiously oriented organizations (ROO).

The seminal ideas of Smith et al. (1983) about OCB shaped a two-dimensional framework including altruism (behavior oriented to help other people) and widespread compliance (behavior in compliance with general rules, norms, and expectations). In a later development, Organ (1988) proposed an expanded model of five dimensions: altruism, courtesy, conscience, civic virtue, and sportsmanship. Different scales of measument were developed for this expanded model (Podsakoff et al., 1990). In a third moment, academics started to build different models, going back to the bidimensional OCB (Williams and Anderson, 1991), focusing on individuals (OCB-I) or organizations (OCB-O). In the work of LePine et al. (2002), the five dimensions were considered again as a set of equivalent indicators. In this line, Hoffman et al. (2007) proposed a model of a single factor correlated with task fulfillment, using OCB in order to evaluate performance at work.

The majority of research done about OCB has concentrated on the individual level and has followed the seminal definition presented by Organ (1988, p. 4): “individual behavior that is discretionary, not directly or explicitly recognized by the formal reward system, and that in the aggregate promotes the effective functioning of the organization.” However, the importance of OCB is based on its collective aggregation, because isolated OCBs have limited impacts in organizations (Organ, 1988). Some studies (see Organ et al., 2005 for a comprehensive review) have pointed out the existence of a positive linkage between collective OCB and organizational productivity. Following this line, collective OCB has turned into a relevant research area. We will highlight the work of academics such as Combs et al. (2006) and Gong et al. (2010) who have positively correlated high-performance work systems (HPWS) with collective OCP through the modulating variable known as collective affective commitment (AC).

Collective OCB refers to the standard way of conduct of the whole group of persons who participate in a given organization (Ehrhart, 2004; Ehrhart and Naumann, 2004). Due to its organizational level, collective OCB has to be boosted and emprirically measured at the collective level (Klein et al., 1994), considering the impact that organizational context may have on observed OCB (Morrison, 1996). In their metanalysis, Combs et al. (2006) argue that high-performance work systems (HPWS), understood as “systems of human resource practices designed to enhance employee’s skills, commitment and productivity in such a way that employees become a source of sustainable competitive advantage” (Datta et al., 2005, p. 136), are positively related to inicators that measure organizational performance or productivity. However, none of the studies which were reviewed by Combs et al. (2006) analyzed if HPWS were linked in any form with collective OCB. In a new study, Sun et al. (2007) analyze the role of OCB in the relationship between HPWS and organizational performance, but they do not conceptualize their constructs as collective OCB, and they do not examine empirically the mechanisms that link HPWS and OCB at the collective level.

Collective behavior is dependent on, among other elements, shared experiences after being exposed to common practices and policies in a given organization (Morgeson and Hofmann, 1999). Employees under the same set of HPWS practices can have a shared collective understanding about their relationship with the organization and, from that point, articulate shared behavioral and actitudinal answers (Schneider, 1987). Affective commitment (AC) is defined as the employee’s positive emotional attchment to the organization (Meyer and Allen, 1991) and has been analyzed using this collective perspective, as a modulating variable between HPWS and an increase in the collective OCB. The main conclusion is this study has been the realization that collective AC is necessary to have a real impact on OCB (Gong et al., 2010). Collective AC can be defined as a “shared mental framework among a collective set of individuals regarding their organization, characterized by feelings of loyalty and a determination to use physical and mental energy to help their organization to achieve their objectives and goals” (Gardner, 2007, p. 7). This definition, in terms of shared belief, is compatible with the one proposed by Bandura (2000) about collective efficacy.

While individual AC refers to a particular employee’s involvement in a given organization (Meyer and Allen, 1991), collective AC considers also the social influence of the group, which can shape the answers of individual employees (Morgeson and Hofmann, 1999). Different research lines of research (Salancik and Pfeffer, 1978; Barsade, 2002; Coté, 2005) coincide in considering that AC emerges from the social processes of interaction inside the group of employees and also from the common exposure to contextual factors in the organization. In that way, AC can be seen as a shared actitudinal answer build by the members of an organization.

## Conceptual Framework to Link the Organizational Citizenship Behavior and Social Accounting

In many cases, academics have used social exchange theory to conceptualize the effect of HPWS in collective OCB through the modulating effect of collective AC (Blau, 1986). Social exchange theory is based on the equilibrium between resources that are put in place in the organization and the results that are obtained (Blau, 1986). It is supposed that participants will follow the reciprocity principle. This means that the receiver will reciprocicate to the part that lends the resources (Gouldner, 1960). The relationship between employees, volunteers or professionals, and the organization considers the interchange of incentives in accordance with the inputs put in place by employees (Tsui et al., 1997). In profit-oriented organizations, incentives are given through HPWS, and employees are thought to correspond with AC and OCB toward the organization. As stated by Morrison (1996, p. 506), “rewards that are based on company-wide performance will mitigate against the quid pro quo mindset inherent in economic exchange relationships, while laying the foundation for social exchange.” In a terminology closer to stakeholder theory, it is possible to affirm that there is shift from transactional to relational behavior (Freeman and Ginena, 2015). This thinking can be traced back to Aristotle (n.d., Nicomachean ethics: 1132 b21), where reciprocity (*antipeponthós*) refers to to the social interactions that keep alife the activity of the *polis*. This kind of reciprocity encompasses all kinds of market activities and even the virtue of friendship (*philia*). This understanding of reciprocity goes beyond the mere interchange of goods or services (Bruni, 2010). On the other hand, HPWS offer employees several social incentives, such as acknowledgement, prestige, growth in the organization, equality in treatment, and empowerment (Morrison, 1996).

### Understanding Organizational Citizenship Behavior From the Needs of Others

The study of OCB from the perspective of social exchange has been solidly developed (Lavelle et al., 2007). However, it does not explain completely why employees and other stakeholders develop OCB behavior. Furthermore, in many ROOs, it would be misguided to propose that the commitment of collaborators is directly linked to the goods and services they receive. As a consecuence, it is desirable to develop and consider theoretical perspectives that may extend the reasons of OCB beyond the mechanisms of mere social exchange (Lavelle, 2010).

OCB is linked with the voluntary behavior of employees beyond their specific duties. OCB has the potential of enhancing organizational performance (Organ, 1988). Considering that this kind of behavior is not compulsory and is not rewarded, then why do employees participate in OCB? Traditionally, researchers have argued that relationships of social exchange derived from positive working atitudes such as affective commitment, equality, and support motivate the citizenship behavior of employees. This means that it is more likely that employees and collaborators could “go beyond” contractual conditions when they feel that they are supported and empowered by their organizations. However, if we explain OCB in this way, it is still an exchange that keeps the equilibrium between inputs and outputs given and generated by individuals or groups of individuals inside an organization (Chahal and Mehta, 2010). This argumentation is even more limited if we want to explain the OCB of stakeholders participating in altruist organizations, where rewards for collaborators are not the key motivational factor to explain their implication (Clary et al., 1998). Posibly, at least in the case of altruist and religious organizations, we should widen the reciprocity theory to a gratouitousness-based theory, aligned for example with the encyclical *Caritas in Veritate* (Retolaza et al., 2019), where the principle of gratuity stands out as overcoming reciprocity (CIV 34; 36 y 38).

Many authors analyzing OCB consider reciprocity as a limited explanation for OCB (Gilbert, 1989; Hurley, 1989; Sugden, 1993; Tuomela, 1995, 2002). Although reciprocity is necessary to build social norms and to generate trust (Bruni and Sugden, 2000), it is not the only kind of relationship that can be established between two economic agents. In the non-profit sector and in religious organizations, motivation of collaborators is not linked mainly with rewards to those collaborators, but with the value that will be generated for other people (usually people in need), from whom no remuneration is expected, not even emotional (Clary et al., 1998).

**Figure 1:**
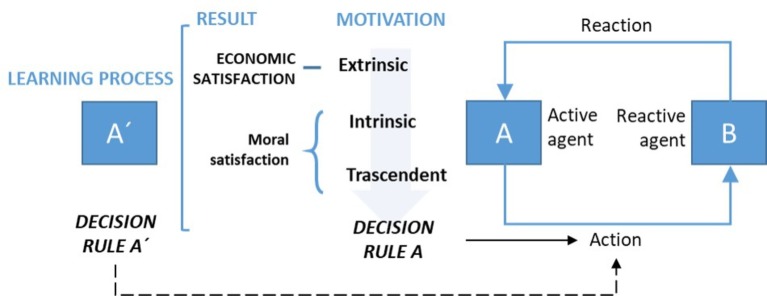
Learning-action-motivation process. Source: own elaboration based on Pérez López (1991).

The theory of human action (Pérez López, 1991) presents a wider understanding of human motivation in comparison with the traditional *homo economicus* model. Egocentric motivations, the ones that are at the core of the traditional economic theory, are only a particular case of the whole theory of human action. This kind of motivation, called extrinsic motivation in this model, consists on achieving a result able to maximize the interest of the active agent. At the same time, the model proposes to take into account two additional sources of motivation. The first one is the motivation based on personal learning (called intrinsic motivation). The second one is the concern for the reactive agent (called transcendent motivation). This last motivation is not present in the traditional economic theory. This motivational complexity demands a shift from transactional motivation (extrinsic) to relational motivation (linked with transcendent motivation) (see Figure 1).

According to the model, the extrinsic level will determine the economic satisfaction of the interaction between agents, whereas intrinsic and transcendent results will determine the moral satisfaction of the person. The model points out that the learning process (including the level of moral satisfaction achieved) produces changes in the active subject, making possible a change in the decision-making over time. On the other hand, the passive agent also achieves a degree of moral and economic satisfaction, being able to change his/her decision-making rule in the same or contrary direction of the active agent. In the first case, and if both are positive, virtuous circles among agents will be generated. In any other circumstance, it may be possible that future interaction could be blocked, or at least made in a vicious circle dominated by the lack of trust.

The model of Pérez López (1991) is consistent with different studies about the behavior of collaborators, where three motivational dimensions are identified: learning, understanding, and achieving social impact (Clary et al., 1998; Clary and Snyder, 1999). The first dimension can be linked with extrinsic motivation, the second with intrinsic motivation, and the third with the transcendent one. In the work of Okun et al. (1998), the key motivational component is associated with moral satisfaction. Furthermore, Frank (2004) was able to monetize (in terms of opportunity cost) transcendent motivation. The theory of human action fits also in the model designed by Barrett (2006, 2013). This last model could be useful to investigate transcendent motivation in the framework of a given organization.

### Organizational Citizenship Behavior and Transcendent Motivation

**Figure 2:**
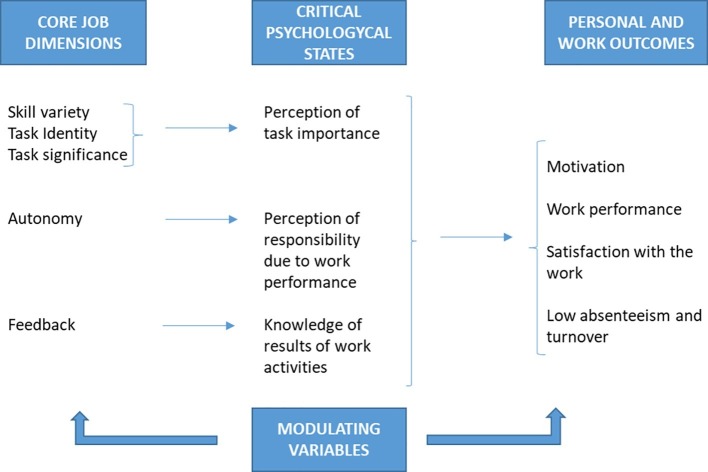
Job dimensions, psychological states, and work outcomes. Source: Own elaboration based on Hackman and Oldham (1976).

In altruist organizations, being religious-oriented or not, it seems clear that transcendent motivation should be a key aspect of collaborators working for them. At the same time, it seems that HPWS, focused on material incentives, can complement economic motivation, but it will have almost no impact on transcendent motivation. Considering that in the organizational context, transcendent motivation is fed back by the results linked with the porpuse of the organization, the degree of this achievement will be importance to determine the moral satisfaction of collaborators and, as a cosecuence, the OCB given to the organization. It is expected that the achievement of objectives related to transcendent motivation will produce moral satisfaction in people (see Figure 2). With this idea in mind, usually social and religious organizations try to circulate among stakeholders’ information about their activities and main results. In this sense, it could be proposed that in the case of sharing systematic positive results about the achievement of the main aim of the organization, this information could enhance moral satisfaction of collaborators, increasing their individual and collective OCB and boosting the organizational performance around its main aim. This proposal could be articulated as a set of interconnected propositions.

It seems possible that information given by social accounting (SA) increases moral satisfaction (MS) of people participating in the organization. If the proposed model of human action were adequate, the increase of moral satisfaction (MS) will be proportional to the transcendent motivation (TM) of the person. And it would be assumed that an ahument of moral satisfaction will increase OCB (both individual and collective). At the same time, the rise in OCB (individual and collective) will increase the high-performance work systems (HPWS) of the organization. Completing the virtuous circle, the rise in the HPWS of the organization will increase moral satisfaction (MS) in participants.

**Figure 3:**
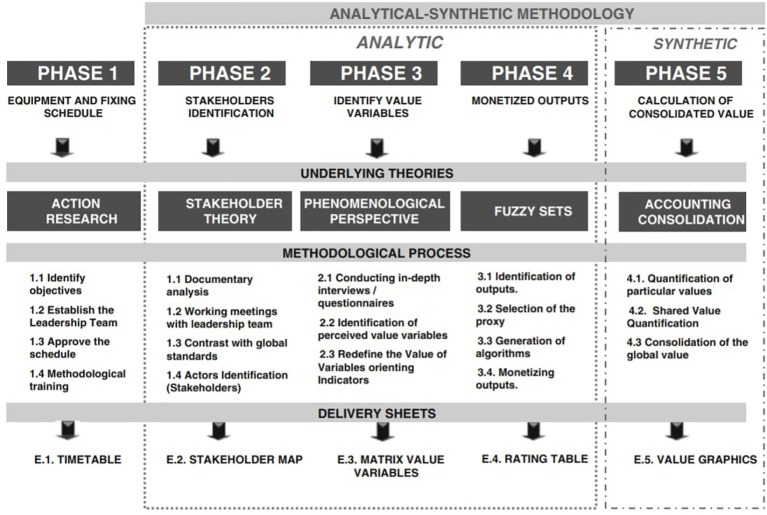
The process of social accounting: SPOLY development process. Source: Retolaza et al. (2016, p. 41).

The joint analysis of all hypotheses demands data from several years. However, in the case under study (the diocese of Bilbao), there is information only about 1 year. Due to that fact, this work will limit its focus on the study of the relationship between the information provided by social accounting and the increase in moral satisfaction of the boards of directors existing in the diocese. Following the model of job characteristics developed by Hackman and Oldham (1976), it is possible to identify five different characteristics: skill and task variety (V), task identity (I), task significance (S), autonomy (A) in the decision making process, and feedback (F) received by the employee or collaborator about his/her efficiency and performance at work. Social accounting is able to answer to the last one (F). In the case of the diocese of Bilbao, this feedback can be given at the global level and also at the level of specific areas. At the same time, social accounting is relevant for identity (I) and task significance (S). In the first case, social accounting helps employees and collaborators to understand their tasks as a part of something bigger, the objective of the firm. In the second place, social accounting identifies the impact of the collaborators’ work (as a part of the whole set of actions of the firm) on the rest of stakeholders and society. This process of feedback can achieve high positive impacts on collaborators that, otherwise, would not identify properly their contribution to the mission of the company, or collaborators with doubts between efficiency and productivity at work. This last ambiguity problem is common in religious or social organizations (see Figure 3).

The aforementioned five dimensions (see Figure 3) do not act directly, but modulated by three basic psychological states: experienced importance of the work, experienced responsibility for outcomes of the work, and knowledge about the results achieved. Combining the five dimensions, it is possible to build the Motivating Potential Score (MPS), as follows:

$$MPS=\left[\left(V+I+S\right)/3\right]*A*F\text{.}$$

As MPS increases, motivation and satisfaction at works for collaborators will be higher. This score can be combined with results coming from social accounting in order to increase moral satisfaction of collaborators at work and their motivation. However, this theoretical formulation has not been tested on religious organizations or non-governmental organizations, which may have different motivational mechanisms in comparison with for-profit organizations. Nevertheless, the introduction of social accounting allows the capture of non-market value generated by organizations and makes possible to contrast the Motivating Potencial Score (MPS) as a modulating variable to obtain OCB.

## Case Analysis: the Diocese of Bilbao

The Diocese of Bilbao decided in 2018 that social accounting could be a useful instrument to measure the social value generated by the diocese and presented it to all its stakeholders and society as a whole. Four different kinds of value were identified: market value, non-market value, emotional value, and spiritual value. Social accounting has focused on the first three, because there is not still any contrasted methodological procedure to measure spiritual value. The first step of applying social accounting to the diocese of Bilbao was to map the perimeter of the diocese, identifying all organizational structures belonging to it. The process started with the educative centers of the diocese, because they were the easiest to be analyzed.

The diocese has five schools, six “ikastolas” or schools which use only the Basque language with students, and five vocational centers distributed in different locations in the Biscay province (1.2 million inhabitants) where the Bilbao metropolitan area is located. Those 16 educative centers employ 1,000 teachers which serve around 10,000 students. Their turnover is about 60 million €, with a positive result of 1–3 million € depending on the year.

All 16 educative centers were created to respond to local needs. In addition, the five vocational centers had the aim of offering vocational training to young people living in industrial areas in crisis, so that finding a job could be a real possibility for students. In all the centers, humanistic principles and civic values based on Catholic Social Thought are encouraged. Some of the centers have the golden Q of the EFQM (European Foundation Quality System) quality system.

The aim of the educative centers is to offer to students and families excellent education, while proposing religious principles and values as part of the integral education of the person. In the centers of vocational training, there is an additional challenge: connect this kind of education with the local labor market, so that students could find a job in a short time.

The reasons of the diocese to initiate this process of social accounting could be summarized in the answers given by the team leader of the project, Jose Joaquin Moral, during a semistructured interview. Mr. Moral explained that “as a general idea, our bishop was interested in measuring the social value that was generated by all the organizations of the diocese of Biscay province and not only *Caritas*, but also parishes, educative centers, the museum, radios, the library, and others.” When Mr. Moral was asked about the interest to monetize the value created by particular entities belonging to the diocese, he answered that “at the particular level, the diocese wanted to measure the social value created by two different vocational centers, one located in a very complex and deteriorated social and economic area, and other located in an area with well structured families and very small poverty rate. The results that were achieved convinced the Basque government (in charge of funding the regional educative system) to increase the funding for the first center, because each euro invested in that center created 2.8 euro to society.” “In other cases, educative centers use social value to know their contribution to different stakeholders and decide if that contribution is balanced or not. In that way, they can introduce changes in their strategic planning, or decide about the investments that are needed.”

The names of the 16 educative centers which have participated in the social accounting project are: Arrantza Eskola, Artzandape, Avellaneda, Begoñazpi, BeraKruz, Iparragirre, Maria Biterko, Otxarkoaga, Sagrado Corazon, San Felix, San Fidel, San Juan, San Viator, Somorrostro, Txomin Aguirre, and Zulaibar.

### Methodological Process

The methodology to be used was well defined before the starting point of the project of the Diocese of Bilbao (Retolaza et al., 2015, 2016) and had been applied to different organizations as hospitals (San-Jose et al., 2019), but always at the individual level. In this case, the project encompassed 16 organizations, creating a potential problem of scalability. In order to minimize the use of resources, the Diocese of Bilbao (in collaboration with the University of Deusto and the non-profit consulting organization GEAccounting) designed an innovative process to consider the 16 entities minimizing time and resources through the use of social accounting (Retolaza et al., 2016).

Following the process, the Diocese started to work with a pilot group of four centers: Begoñazpi, San Felix, Sagrado Corazon, and Somostro. Those centers had more human and economic resources available in comparison with the others. Each of those four centers developed autonomously phases 1, 2, and 3 (see Table 1), obtaining the map of stakeholders and the variables that created value in each center. Combining the results obtained by the four centers, a standardized map of stakeholders and the matrix of value variables valid for the whole set of 16 centers was built. In this way, 19 variables of value that could be valid for all centers were identified. At the same time, the proxies needed to quantify and monetize those variables (phase 4) were identified in the same way for the four pilot centers.

**Table 1:** Consolidated values of social accounting for the 16 educative centers of the Diocese.

	Society	Government	Suppliers	Workers	Social entities	Investors	Users
Added Value	47.012.447 €	16.502.082 €	0 €	26.020.635 €	0 €	0 €	0 €
Value Mobilized (I)	3.772.574 €	2.578.177 €	1.648.190 €	497.792 €	0 €	290.478 €	0 €
Value Mobilized (II)	836.250 €	690.537 €	272.103 €	104.212 €	0 €	23.268 €	0 €
Social Value	0 €						
**Market Social Value [MSV]**	51.621.271 €	19.770.796 €	4.161.195 €	26.622.638 €	0 €	313.746 €	0 €
**Non-Market Social Value [NMSV]**	70.184.825 €	66.629.081 €	0 €	8.348.656 €	3.814.642 €	0 €	66.629.081 €
**Integrated Social Value [ISV]**	121.806.096 €	86.399.877 €	4.161.195 €	34.971.294 €	3.814.642 €	313.746 €	66.629.081 €
EMOTIONAL VALUE	*28.103.469 €*						
**Socio-Emotional Value [SEV]**	***149.909.565 €***						

Once completed the final phase (phase 5, see Table 1) for the four pilot centers, the process was implemented for the remaining 12 centers. This was done in two sessions using the standardized map of stakeholders and matrix of value variables. In the first session, the standized matrix was explained and contrasted with the directors of the 12 centers. If any variable of the matrix did not apply to a specific center, then the output for that variable in the case of that given center would be 0. If any of the remaining 12 centers could identify any additional variable, that center was encouraged to add that variable in the matrix. In the end, only one center decided to add a new variable linked with activities related with development cooperation, a very specific topic that took place only in that center. Once all variables were understood and accepted, the 12 centers were asked to identify the outputs generated in relation to the aforementioned variables (see phase 4 in Table 1). In the second session, outputs were given the monetary values agreed in the pilot group. With this last step (see phase 5 in Table 1), the process of social accounting for the whole set of 16 entities was completed. In a brief period (less than 1 month) and with a reasonable effort in time and resources (two sessions with directors), the process for the remaining 12 entities was completed.

The process developed the individual social accounting for each of the 16 centers and also was able to build the consolidated social accounting for the whole set of 16 centers. In the next section, those results will be analyzed. In addition, its use in order to increase moral satisfaction of stakeholders and the OCB of collaborators will be studied.

### Discussion of Results

The consolidated social accounting for the 16 centers generated the results explained in Table 1. Rows identify the different typologies of value: first, Market Social Value (MSV), which is the one that occurs in transactions with price. It is included in the accounting. This value is divided into three different typologies: (1) added value, consisting of the difference between purchases and sales, that corresponds to the generate value provided by the organization; (2) value mobilized to suppliers of exploitation (I), and (3) value mobilized to suppliers of investment (II), both through purchasing power. Second, Non-Market Social Value (NMSV), also called specific social value, which is the one that is transferred to the margin of a price system, and therefore is not included in the accounting information. Finally, the Emotional Value (EV), which corresponds to the Affective commitment of Meyer and Allen (1991). Integrated social value (ISV) is the sum of market value (MSV) and non-market value [ISV = MSV + NMSV]; while the Socio Emotional Value (SEV) is obtained by adding the integrated social value and the emotional value [SEV = ISV + EV] (for a comprehensive analysis of methodological steps see Retolaza et al., 2015, 2016). Columns show the value generated by each stakeholder: public administration, suppliers, employees, social entities, inversors, and users/clients. The first column, society, gathers the consolidated value considering all stakeholders (see Table 1).

The information gathered in Table 1 is useful to communicate the value generated by the 16 centers for society, distributed among stakeholders. At the same time, that information may have an impact on the moral satisfaction of collaborators and other stakeholders that are engaged in the activity of those centers. In order to analyze that impact, it is necessary that stakeholders could understand the meaning of the information of Table 1. One way of making easier this understanding is through the use of ratios (Barnes, 1987; Williams and Dobelman, 2017). In any ratio, there is always a numerator and a denominator. In the case of social accounting, the numerator will be the generated value and a potential denominator could be the total budget. This ratio is the result of comparing social value (output) with budget (input) and is called Social Value Added Index (SVAI). This index has been used recently in other cases (Lazcano et al., 2019; San-Jose et al., 2019). In short, SVAI compares the social value generated by an organization with its budget. If both magnitudes are equal, then SVAI will be 1. This value could be used as a reference in order to understand real values achieved by organizations, such as the 16 centers under study in this case.

However, in all economic activities, it is supposed that some form of added value is going to be generated. In this line, it would be possible to build a sector-based expected SVAI (SVAI_s_), in order to establish a standard SVAI result by sector. In that way, it could be possible to obtain the social OCB of an organization as the difference between SVAI and SVAI_s_, as follows:

$$OCB=\mathrm{SVAI}-{\mathrm{SVAI}}_{s}$$

In the case of this study, the ratios that have been calculated are the ones in Table 2. The Economic Retun Ratio refers to the value generated for each euro of the budget used. The Social Value Added Index (SVAI) refers to the social (non-market) value generated for each euro of the budget used. The Integral Social Return Ratio refers to the non-market and market value obtained for each euro of the budget used. And finally, the Socio-Emotional Return Ratio refers to the sum of the Integral Social Value and the Emotional Value obtained for each euro of the budget used.

**Table 2:** Social efficiency ratios for the 16 centers of the Diocese.

Ratio	Result
Economic retun ratio	1,02
Social value added index (SVAI)	1,39
Integral social return ratio	2,00
Socio-emotional return ratio	2,59

In the Basque region, education is an activity heavily dependant on funding coming from the regional administration (the Basque government). All state-owned and most of private education centers receive public funding. All educative centers are non-for-profit organizations. Based on this situation, it is reasonable to estimate that SVAI_s_ in the Basque education system could be similar to 1, indicating that there is an equivalence between the value generated and the budget used to generate it. It is a purely theoretical approximation of equivalence between inputs and outputs; or what is the same, the value of the services generated is equal to their cost. This gives us a minimum reference value, where the value generated for the company must be equal to or greater than 1, to justify the adequacy of the resources used. It could be that in practice this value is higher or lower, but this hypothesis is aligned with previous studies that have calculated shadow prices to calculate the social value generated by the education sector in the Basque region (San-Jose et al., 2019). Under this premise, the calculated SVAI for the education centers of the Diocese of Bilbao is reflecting that the value generated by them is a 39% higher than the economic resources utilized. This value (39%) could be used as an approach to the OCB of the 16 education centers of the Diocese, following the expression OCB = SVAI − SVAI_s_.

At the same time, Table 3 highlights that the SVAI generated in each education center is different. This result is consistent with the idea that OCB is organization-specific and depends on idiosyncratic characteristics of each center.

**Table 3:** SVAI of each education center of the Diocese.

CENTRO 1	CENTRO 2	CENTRO 3	CENTRO 4	CENTRO 5	CENTRO 6	CENTRO 7	CENTRO 8
1,496	1,613	1,400	1,380	1,686	1,349	1,298	1,000
CENTRO 9	CENTRO 10	CENTRO 11	CENTRO 12	CENTRO 13	CENTRO 14	CENTRO 15	CENTRO 16
2,064	1,349	0,342	1,555	1,005	1,048	0,910	0,991

Considering both Tables 2, 3, it is possible to measure two differente OCBs. This means that it is possible to build an aggregate and an individual OCB. If we consider the SVAI calculated for the whole set of education centers of the diocese (see Table 2), the result is that this aggregate SVAI indicates a higher performance (>39%) in comparison with the standard SVAI for the Basque education sector (SVAI_s_ = 1). This result is compatible with a higher level of stakeholders’ moral satisfaction in the centers of the diocese; it is visualized based on the interviews with the directors of the centers. Besides, individualized SVAI can be a tool for benchmarking. In this case, some individual centers exceed the score of 1, while others have achieved results under 1. Depending on individual SVAIs (see Table 3), impacts on stakeholders’ moral satisfaction will be different.

After this análisys, directors of all 16 centers answered a questionnaire about the process and all of them stated the uselfullness of social accounting in order to be aware of the social value generated by their centers. In addition, they underlined that the results of the study allowed them to understand in a wider framework the tensions between answering the needs of stakeholders (efficacy) and/or achieving the management goals of efficiency. Directors highlighted the utility of social accounting in three major fields: external communication mainly to the Basque government (who is the main provider of funding for the centers) and in relation to other stakeholders; information for management and strategic planning; empowerment of stakeholders, specially collaborators.

One of the main results of this work is that SVAI could be a proper indicator for collective OCB in an organization or groups of organizations. Its knowledge by collaborators and stakeholders is useful to highlight the social value generated by a given organization. At the same time, collaborators, also in the case of entities belonging to the Diocese of Bilbao, may develop an alignment between motivation, moral satisfaction, and the overall performance of the organization. In many religious and social organizations (as in the case of the Diocese), collaborators are not only motivated by the classic HPWS. A transcendent motivation is also important in these cases. At the same time, it is posible to consider emotional value as an indicator to measure the difference between the efforts put in place (in time, resources, or any other input) and the value that has been created. In there is an equilibrium between efforst and value and then emotional value would have a neutral score (3 out of 5 points in a scale from 0 to 5). Emotional value would have a positive score (4 or 5 point) if the created value is considered higher than all inputs involved and a negative one otherwise. This last case could make volunteers to abandon their tasks in the organization.

## Conclusions

This research has connected concepts which were not traditionally linked in the literature, such as OCB and moral satisfaction, in a single framework. In addition, this research has inserted the relationships between OCB and moral satisfaction in the systemic functioning of the theory of human action. This last step has introduced the concepts of learning and transcendent motivation in the analysis of motivational processes inside organizations. This is a fundamental change in comparison to the homo economicus anthropological model, which is at the base of many works in the field of motivation in organizations.

Besides, a linkage between OCB (more related with personal efforts at the workplace) and HPWS (focused in the global organizational result) is stablished.

In addition, this work has made arguable the proposition of SVAI as an indicator for high-performance work systems (HPWS) and, indirectly, also for OCB. At the same time, the use of emotional value is proposed as an indicator for moral satisfaction. In all cases, social accounting has served as a measurement tool. In this line, the example of the educative centers of the diocese of Bilbao has been presented.

Precisely in the case of the diocese, social accounting and SVAI have been presented to directors of the educative centers as tools for the empowerment for collaborators, and as catalyzers to align employees’ actions with the strategy of those centers. However, it has not been possible to determine a positive linkage between OCB (measured through SVAI) and moral satisfaction (measured through emotional value). The relationship between these two variables (OCB and moral satisfaction) seems to be a complex one. As expressed in previous sections, transcendent motivation of collaborators could be a nexus between the two. At the same time, MPS (Motivating Potential Score) has been presented as a potential mechanism able to explain how through social accounting it is possible to transform OCB into moral satisfaction. The creation of a synthetic index (SVAI) and the calculation of similar indexes for each stakeholder could allow the generation o fan aim-based feedback for stakeholders. According to the equation explained in the section “Organizational Citizenship Behavior and Transcendent Motivation,” this feedback should increase the MPS of employees. As long as this feedback achieves an increase in the results and motivation of employees, it could be possible to foster a virtuous circle coherent with the propositions of stakeholder theory (San-Jose et al., 2017). However, in its current development, this is only a theoretical proposal. The empirical studies and contrasts should be developed in future works.

One of the main limitations of this work is the fact that theoretical reflection has been made at the same time as the development of the process to implement social accounting in organizations. This situation has made impossible the introduction of modulating variables, such as transcendent motivation. Two lines of research emerge as key issues in the near future. On one hand, more research is needed to fix de theoretical level for SVAI_s_. On the other hand, the design of a comprehensive process in order to link social accounting with SVAI, HPWS, and OCB could clarify the linkages between these four concepts and foster the understanding of their joint operation, with the final aim of increasing both OCB and moral satisfaction inside organizations; and consequently, the resulting HPWS.

## Data Availability Statement

The datasets generated for this study are available on request to the corresponding author.

## Author Contributions

All authors have jointly developed the article, and to a greater or lesser extent all have participated in the development of the social accounting of the Diocese of Bilbao.

### Conflict of Interest

The authors declare that the research was conducted in the absence of any commercial or financial relationships that could be construed as a potential conflict of interest.
